# Supplementary material for: Robust diagnosis of Ewing sarcoma by immunohistochemical detection of super-enhancer-driven EWSR1-ETS targets
Source: Oncotarget. 2017 Aug 4;9(2):1587–601. doi: 10.18632/oncotarget.20098 (PMC5788584; doi:10.18632/oncotarget.20098)
Supplement: Supplementary file 1 [file oncotarget-09-1587-s001.pdf]

Robust diagnosis of Ewing sarcoma by immunohistochemical detection of super-enhancer-driven EWSR1-ETS targets

SUPPLEMENTARY MATERIALS

For Supplementary Tables 1–5 see in Supplementary Files

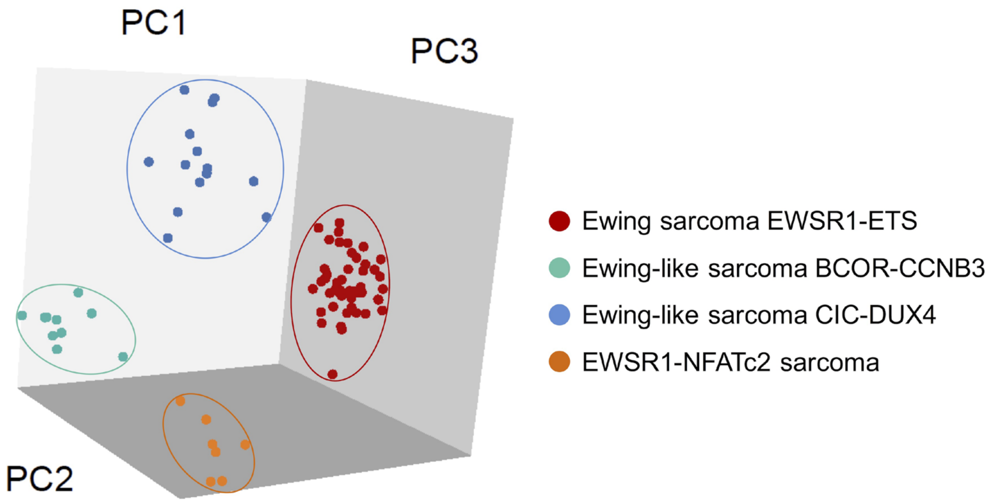

Supplementary Figure 1: Unsupervised PCA of CIC-DUX4-, BCOR-CCNB3-, EWSR1-NFATc2-translocated sarcomas as well as EWSR1-ETS-translocated Ewing sarcomas.
PCA was performed based on 19,702 expressed genes using the statistical language R. Each dot represents an individual sample.

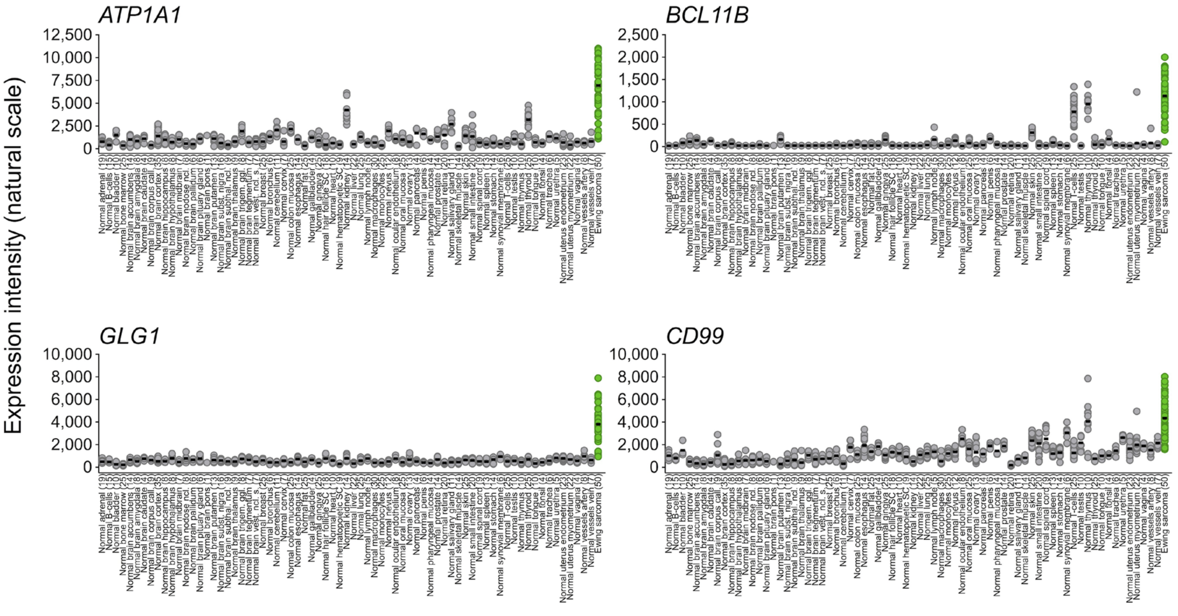

Supplementary Figure 2: Comparison of *ATP1A1, BCL11B,* and *GLG1* mRNA expression in Ewing sarcoma and normal tissues. Microarray data were normalized simultaneously by RMA using custom brainarray CDF files (v19) yielding one optimized probe-set per gene [1]. Accession codes are given in Supplementary Table 5. Ewing sarcoma is highlighted in green color. Horizontal bars represent median expression values. Numbers of analyzed samples are given in parentheses.

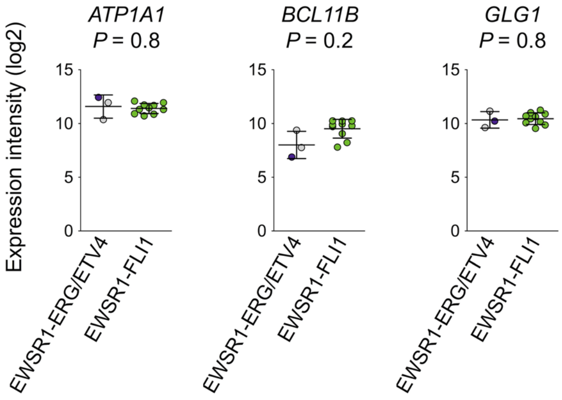

Supplementary Figure 3: *ATP1A1, BCL11B*, and *GLG1* are similarly expressed in EWSR1-FLI1- and EWSR1-ERG/ ETV4-translocated Ewing sarcoma. All microarray data were retrieved from the GEO (accession codes: GSE8596, GSE36133, and GSE70826), generated on Affymetrix HG-U133 Plus2 arrays, and normalized simultaneously with RMA using custom brainarray CDF (v17). Gray dots, EWSR1-ERG; Purple dot, EWSR1-ETV4; green dots, EWSR1-FLI1. Horizontal bars represent mean expression values; whiskers represent the standard deviation.

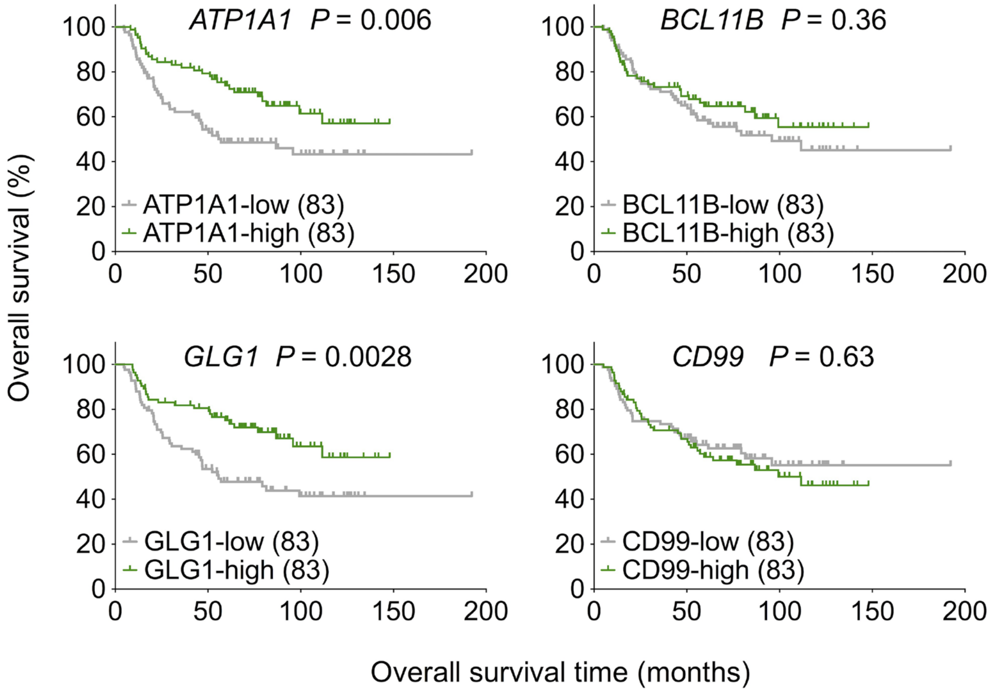

Supplementary Figure 4: ATP1A1 and GLG1 may serve as prognostic biomarkers in Ewing sarcoma. Microarray data of 166 primary Ewing sarcoma tumors were normalized by RMA using custom brainarray CDF files (v20). Samples were stratified into two groups based on their median intratumoral gene expression levels. Significance levels were calculated with a log-rank test.

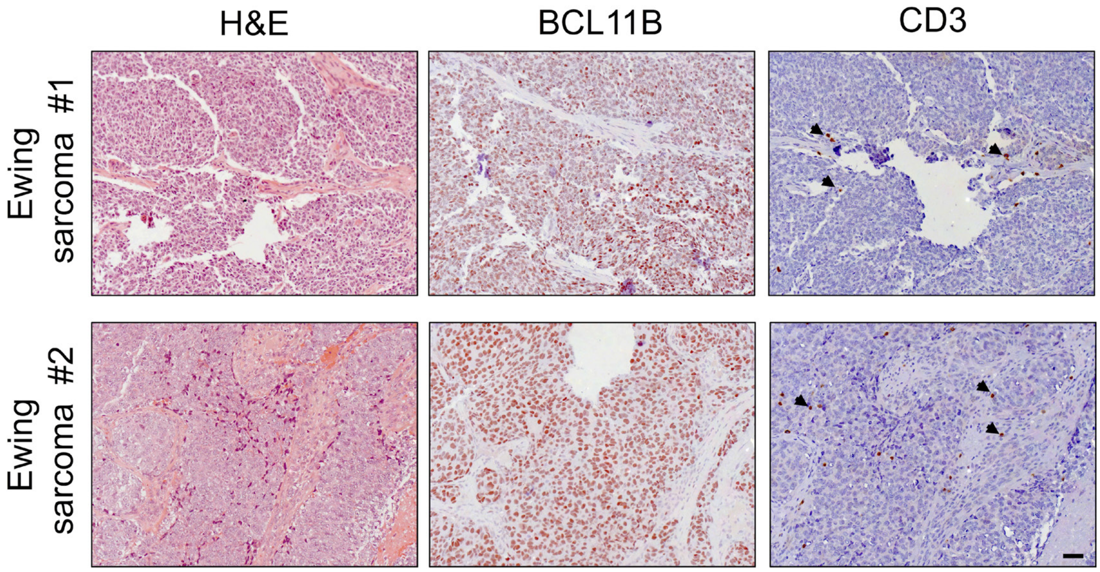

Supplementary Figure 5: Representative images of Ewing sarcoma cases stained for H&E, BCL11B, and CD3. Arrows point at tumor-infiltrating lymphocytes. Scale bar = 100 µm.

Reference

1. Dai M, Wang P, Boyd AD, Kostov G, Athey B, Jones EG, Bunney WE, Myers RM, Speed TP, Akil H, Watson SJ, Meng F. Evolving gene/transcript definitions significantly alter the interpretation of GeneChip data. Nucleic Acids Res. 2005; 33: e175. doi: 10.1093/nar/gni179.
